# Correction: Investing in entropy: the strategy of cucurbit[*n*]urils to accelerate the intramolecular Diels–Alder cycloaddition reaction of tertiary furfuryl amines

**DOI:** 10.1039/d4sc90121e

**Published:** 2024-06-28

**Authors:** Karen de la Vega-Hernández, Marcos G. Suero, Pablo Ballester

**Affiliations:** a Institute of Chemical Research of Catalonia (ICIQ-CERCA), The Barcelona Institute of Science and Technology (BIST) Avgda. Països Catalans 16 Tarragona 43007 Spain mgsuero@iciq.es pballester@iciq.es; b ICREA, Passeig Lluís Companys 2308018 Barcelona Spain

## Abstract

Correction for ‘Investing in entropy: the strategy of cucurbit[*n*]urils to accelerate the intramolecular Diels–Alder cycloaddition reaction of tertiary furfuryl amines’ by Karen de la Vega-Hernández *et al.*, *Chem. Sci.*, 2024, **15**, 8841–8849, https://doi.org/10.1039/D4SC01816H.

The authors regret that a project number was omitted in the acknowledgments of the original manuscript. The complete acknowledgments are as shown below.

This work has received funding from the European Union's Horizon 2020 Research and Innovation Programme under the Marie Skłodowska-Curie grant agreement No. 801474, the Ministerio de Ciencia, Innovación y Universidades/Agencia Estatal de Investigación (MICIU/AEI/10.13039/501100011033; PID2020-114020GB-100; PID2022-140286NB-I00), the Severo Ochoa Excellence Accreditation 2020–2023 (CEX2019-000925-S) and AGAUR (2021SGR00851). We also thank CERCA Programme/Generalitat de Catalunya and ICIQ Foundation for financial support.

The Royal Society of Chemistry apologises for these errors and any consequent inconvenience to authors and readers.

